# Accuracy of conventional identification methods used for Enterobacteriaceae isolates in three Nigerian hospitals

**DOI:** 10.7717/peerj.2511

**Published:** 2016-09-28

**Authors:** Christiana Jesumirhewe, Peter Oladejo Ogunlowo, Mitsan Olley, Burkhard Springer, Franz Allerberger, Werner Ruppitsch

**Affiliations:** 1 Department of Pharmaceutical Microbiology, Igbinedion University Okada, Okada, Edo state, Nigeria; 2 Department of Medical Microbiology, Igbinedion Univesity Teaching Hospital, Okada, Edo state, Nigeria; 3 Austrian Agency for Health and Food Safety (AGES), Institute of Medical Microbiology and Hygiene, Vienna, Austria

**Keywords:** Enterobacteriaceae, Bacterial identification, MALDI-TOF MS

## Abstract

**Background:**

Enterobacteriaceae are ubiquitously present in nature and can be found in the intestinal tract of humans and animals as commensal flora. Multidrug-resistant Enterobacteriaceae are increasingly reported and are a threat to public health implicating a need for accurate identification of the isolates to species level. In developing countries, identification of bacteria basically depends on conventional methods: culture and phenotypic methods that hamper the accurate identification of bacteria. In this study, matrix-assisted desorption ionization-time of flight mass spectrometry (MALDI-TOF MS) technique was compared to conventional identification techniques.

**Materials and Methods:**

In total, 147 Enterobacteriaceae isolates were collected from March to May 2015 from three medical microbiology laboratories of hospitals in Edo state, Nigeria, after being tested according to the individual laboratories standard operating procedures. All isolates were stored at −20°C until tested centrally by MALDI-TOF MS.

**Results:**

One hundred and forty five (98.6%) isolates had a MALDI Biotyper best score > or =2.0, indicating a secure genus and probable species identification; and 2(1.36%) isolates had a best score <2.0 indicating probable genus identification. Isolates with best scores of > or =2.0 comprised nine genera and 10 species, respectively. A total of 57.2% and 33.1% of isolates identified had agreement between MALDI-TOF MS and conventional techniques for identification at genus and species level, respectively, when analyzing bacteria with MALDI Biotyper best scores > or =2.0.

**Conclusion:**

The results of our study show that the applied conventional identification techniques for Enterobacteriaceae in the investigated Nigerian hospitals are not very accurate. Use of state-of-the-art identification technologies for microorganisms is necessary to guarantee comparability of bacteriological results.

## Introduction

Bacterial identification to the species level is a very important aspect of microbiology. Conventionally, this relies on phenotypic characteristics, genotypic traits and immunological (serological) analysis of microorganisms. Commercial identification systems like API (bioMerieux) or Microbact 12E/24E (Oxoid) are used for this purpose in some clinical settings; however, resource restrictions limit their application (Massonet et al., 2004; Panda et al., 2014). In tropical and developing countries like Nigeria, bacterial identification is traditionally achieved by carrying out labour intensive and time consuming homemade biochemical assays which may not be useful in a situation where results are urgently needed for medical diagnosis (Cheesebrough, 2000; Abdessalam et al., 2010; Iroha et al., 2011). In addition, not all microorganisms are reliably identified by biochemical methods. These methods often give wrong results in an unacceptable rate (Ayeni et al., 2015). In developing countries, culture media like MacConkey agar, chocolate agar, blood agar, are usually used in medical laboratories to demonstrate the presence of Enterobacteriaceae isolates in clinical samples. After the growth of the isolates on the culture media, Gram staining is also used to aid identification. Biochemical tests, for example Beta-glucoronidase test, citrate utilization test, urease test, indole test, are the basic tests used for Enterobacteriaceae identification in Nigerian hospitals (Cheesebrough, 2000).

Matrix-assisted laser desorption ionization-time of flight (MALDI-TOF) mass spectrometry is a rapid and accurate method for identification of microorganisms (Bizzini & Greub, 2010; Iroha et al., 2011; Panda et al., 2014; Ayeni et al., 2015) with a high degree of specificity and sensitivity. Classification and identification of microorganisms using the MALDI Biotyper systems are based on proteomic fingerprinting using high-throughput MALDI-TOF mass spectrometry. In MALDI-TOF MS, crystallized sample material is ionized by short laser pulses. The ions are accelerated and their time of flight is measured in a vacuum flight tube (Wieser et al., 2012). Biomolecules are separated by the mass-to-charge ratio in the flight tube. Each biomolecule generates a distinct signal hence molecular signatures can be detected for different microorganisms which is used for comparison with the stored reference spectra thereby providing sample identification. The earliest application of MALDI-TOF MS for identification of bacteria dates back to 1975 (Anhalt & Fenselau, 1975). Reports on the application of MALDI-TOF MS in the clinical microbiology laboratory have been increasing for the past decade (Seng et al., 2009; Bizzini & Greub, 2010; Iroha et al., 2011). However, limited reports on its application in developing countries exist. A major advantage of MALDI-TOF MS is the rapid turnaround time used in accurate identification of the organism compared with conventional techniques. It requires only a small amount of a single colony of microorganisms and has a simple sample preparation process (Panda et al., 2014).

The phenotypic and immunological methods used in tropical and developing countries like Nigeria for identification of microorganisms are often of limited number, thereby resulting in misidentification or inaccurate identification of microorganisms. In this study, the accuracy of local cultural, morphological and biochemical techniques for the identification of Enterobacteriaceae isolates was compared to MALDI-TOF MS as gold standard.

## Materials and Methods

### Bacterial isolates

One hundred and forty seven Enterobacteriaceae isolates of clinical origin were collected from three different hospitals including University of Benin Teaching Hospital, Central Hospital Benin and Igbinedion University Teaching Hospital Okada all in Edo state, Nigeria from March to May 2015. One hundred and twenty four (84.4%) out of the 147 isolates were collected from the University of Benin Teaching Hospital, Benin City. The conventional methods adopted for the identification of the bacterial isolates were as described by Cheesebrough (2000). These methods for identification were carried out on all isolates in the three separate laboratories in the hospitals from where the isolates were obtained. The results from the three separate laboratories were from routine clinical microbiology service.

### Gram staining

Gram staining classifies bacterial isolates into Gram-positive and Gram-negative on the basis of differential interactions of Gram reagents with the varying cell wall components of these two groups of bacteria. All isolates were Gram stained to aid their identification.

### *Beta*-glucoronidase test

This test was used for identification of *E. coli*. A dense milky suspension of the organism to be tested was prepared in a small tube containing 0.25 ml of saline. One tablet of 4-nitrophenyl-*β*-D-glucopyranosiduronic acid (PGUA)for detection of *β*-glucoronidase activity was added to the tube. A stopper was placed in the tube and the tube agitated vigorously for a few seconds. The tube was incubated afterwards at 35–37°C for 4 h. The development of a yellow colour in the supernatant indicates a positive test

### Citrate utilization test

This test works on the ability of an organism to utilize sodium citrate as the sole carbon source for growth and metabolism which results in the alkalinization of the medium. The Koser’s citrate medium used contains sodium citrate as the only carbon source and bromo-thymol blue as indicator. The utilization of citrate for bacterial growth results in the production of alkaline by-products which raises the pH of the medium and eventually causes a colour change from green to blue. A 24 h-old culture of the test isolate was inoculated into a 3 mL aliquot of the medium. This was incubated at 37°C for four days and observed daily for degradation of citrate leading to alkalinisation of the medium, which is indicated by the pH indicator bromothymol blue changing colour from green to deep blue along with the growth of the organism, to indicate a positive result. A negative test reaction was shown by no-change in colour, without any growth of the isolate.

### Urease test

The test isolates were innoculated in the tubes containing slants of Christensen’s urea agar medium and incubated at 35°C for 72 h. The tubes were examined over 12 h and after 24 h. Urease production lead to the hydrolysis of urea to ammonia which increases the pH as indicated by colour change in the medium from yellow to pink.

### Indole test

Sterile tryptone water medium was inoculated with the test isolate and incubated at 35–37°C for up to 48 h. Kovac’s reagent was then added in 0.5 mL volume to the culture and shaken gently. A red colour in the surface (the alcohol layer) of the medium implied a positive reaction. No-colour change indicates a negative reaction.

### Lactose fermentation test

All Enterobacteriaceae isolates were inoculated on Mac-conkey agar plates and incubated for 18 h at 37°C. The plates were then observed for growth coloration, a pinkish coloration of colonies as a positive reaction.

Tables 1 and 2 summarize data for these *Enterobacteriaceae* isolates and the conventional identification tests results respectively. The conventional identification test results showed *Klebsiella* sp, *Klebsiella oxytoca*, *Enterobacter* sp and *Citrobacter* sp to have closely related identifications. From the results obtained, the indole test and the urease test were used to distinguish *Klebsiella* sp from *K. oxytoca* and *Enterobacter* sp respectively. *Citrobacter* sp was differentiated from *Klebsiella* sp based on its morphology. All isolates were stored at −20°C until tested centrally by MALDI-TOF MS. For MALDI-TOF mass spectrometry analysis, all isolates were plated on Drigalski agar (BD Difco) and incubated for 24 h at 37°C.

**Table 1:** Summarized data of Enterobacteriaceae isolates provided by three Nigerian hospitals.

Hospital	Sample source	Enterobacteriaceae isolates as identified by the hospitals
University of Benin Teaching Hospital, Benin	Urinary catheter tip *n* = 3, Blood culture *n* = 3, Swabs *n* = 27, Urine *n* = 82, Wound HVS aspirate *n* = 1, Synovial fluid *n* = 1, Pleural aspirate *n* = 1, Unidentified source *n* = 6	*Escherichia colin* = 55, *Klebsiella* sp. *n* = 47, *Klebsiella oxytocan* = 9, *Proteus* sp. *n* = 1, *Proteus vulgarisn* = 4, *Citrobacter* sp. *n* = 7, *Enterobacter* sp. *n* = 1 Total *n* = 124
Central Hospital, Benin	Urine *n* = 9, Swabs *n* = 5, Pus *n* = 1	*Escherichia colin* = 3, *Klebsiella* sp. *n* = 9, *Proteus* sp. *n* = 3 Total *n* = 15
Igbinedion Teaching Hospital, Okada	Urine *n* = 4, Swab *n* = 3, Stool *n* = 1	*Escherichia colin* = 3, *Klebsiella* sp. *n* = 5 Total *n* = 8

**Table 2:** Summarized data of Conventional identification tests results.

	RESULTS						
TEST	EC	Ksp	KO	Esp	Pvul	Psp	Csp
Gram-stain	Gram- negative	Gram- negative	Gram- negative	Gram- negative	Gram- negative	Gram- negative	Gram- negative
Morphology	Small rods	Rods	Rods	Rods	Rods	Rods	Small rods
Lactose fermenting	Positive	Positive	Positive	Positive	Negative	Negative	Positive
Indole	Positive	Negative	Positive	Negative	Positive	Negative	Negative
Urease	Negative	Positive	Positive	Negative	Positive	Positive	ND
Citrate	Negative	Positive	Positive	Positive	ND	ND	Positive
B-glucoronidase	Positive	ND	ND	ND	ND	ND	ND

**Notes.**

TITLE EC *Escherichia coli* Ksp *Klebsiella sp* KO *Klebsiella oxytoca* Esp *Enterobacter sp* Pvul *Proteus vulgaris* Psp *Proteus sp* Csp *Citrobacter sp* ND Not determined

### Sample preparation for MALDI-TOF MS

A thin smear from a single colony of a 24 h old culture was deposited in duplicates on a MALDI-TOF sample plate (Bruker Daltonik GmbH, Bremen, Germany). The samples were made in duplicates to test the reproducibility of the instrument. Mucoid samples for example *Klebsiella pneumoniae*, some *Enterobacter cloacae* and *Escherichia coli* isolates which were difficult to identify by depositing a smear of the isolates directly on the MALDI-TOF MS sample plate were pretreated using the ethanol/formic acid extraction procedure following the manufacturer’s instruction (Bruker Daltonik GmbH, Bremen, Germany). For the pretreatment procedure, an isolated colony was transferred by an inoculating loop into a 1.5ml extraction tube containing 300 µl of High Performance Liquid chromatography (HPLC) water. The colony was suspended by pipetting and vortexing for at least 1 min. 900 µl of ethanol was added, tubes were vortexed for at least 1 min and centrifuged for 2 min at 13,000 rpm; the supernatant was discarded and the tubes were centrifuged again at 13,000 rpm for 2 min. Excess ethanol left in the tubes was removed by pipetting before allowing the pellets to air-dry for 5 min. Five µl of 70% formic acid was added to suspend the pellet by pipetting and vortexing before addition of an equivalent volume of acetronitrile. The mixture was centrifuged for 2 min at 13,000 rpm and 1 µl of the clear supernatant was placed onto the MALDI-TOF target plate and allowed to air dry. All samples on the target plate were overlaid with 1 µl of matrix solution (saturated solution of *α*-cyano-4-hydroxycinnamic acid (HCCA) in 50% acetronitrile and 2.5% triflouroacetic acid (final concentration: 10 mg HCCA/ml) and air dried at room temperature.

### MALDI-TOF MS measurement

The MALDI-TOF MS target was subsequently introduced into the MALDI-TOF mass spectrometer for analysis using a microflex MALDI-TOF mass spectrometer (Bruker Daltonik GmbH, Bremen, Germany) with a Flexcontrol software (version 3.0). The spectra were recorded in the linear positive mode (laser frequency 20 Hz, ion source 1 voltage, 20 kV; ion source 2 voltage, 18.4 kV; lens voltage, 9.1 kV; mass range, 2,000 to 20,000 Da). For each spectrum 240 shots in 40- shot steps were collected from different positions of the target spot and analyzed by comparing the collected spectra with a reference database containing a wide variety of medically relevant isolates (Wieser et al., 2012; Ayeni et al., 2015). MALDI-TOF MS results were expressed as proposed by the manufacturer with scores ranging from 0–3 based on the similarities between the observed and the stored data sets which provide information about the validity of the identification: scores ≥ 2.3–3.0 indicates highly probable species identification, scores of ≥2.0 were used for secure genus and probable species identification, a score of ≥1.7 and <2.0 was considered for probable identification to the genus level and scores below 1.7 were considered not to have generated a good identification. The highest score of a match against a spectrum in the database was used for bacterial identification (Panda et al., 2014; Ayeni et al., 2015).

**Table 3:** Identification of human clinical Enterobacteriaceae isolates with MALDI-TOF MS compared to conventional tests (Biochemical Tests).

	MALDI Biotyper score ≥ 2.0			MALDI Biotyper score ≥ 1.7		
Isolates	No tested	Agreement at genus level	Agreement at species level	No tested	Agreement at genus level	Agreement at species level
Enterobacteriaceae	145	83(57.2%)	48(33.1%)	2	1(50%)	1(50%)
*Escherichia coli*	60	48(80%)	48(80%)	1	1(100%)	1(100%)
*Citrobacter spp*	6	0	0	1	0	0
*Proteus spp*	4	3(75%)	0	0	0	0
*P. vulgaris*	4	1(25%)	0	0	0	0
*Klebsiella spp*	61	26(42.6%)	0	0	0	0
*K. oxytoca*	9	4(44.4%)	0	0	0	0
*Enterobacter sp*	1	1(100%)	0	0	0	0

**Table 4:** Data of Enterobacteriaceae isolates misidentified by Conventional test identification methods.

Number	Hospital	Identification	Conventional tests identification	MALDI-TOF MS identification
1	UBTH	1482	*Klebsiella* sp	*E. coli*
2	UBTH	5832	*Klebsiella* sp	*E. asburiae*
3	UBTH	1259	*Klebsiella* sp	*E. asburiae*
4	UBTH	4501	*Klebsiella* sp	*E. cloacae*
5	UBTH	5774	*Klebsiella* sp	*E. cloacae*
6	IUTH	6II	*Klebsiella* sp	*E. coli*
7	UBTH	3461	*Klebsiella* sp	*C. freundii*
8	UBTH	138	*Klebsiella* sp	*S. marcescens*
9	UBTH	8(2)	*Klebsiella* sp	*E. coli*
10	UBTH	3904	*Klebsiella* sp	*E. cloacae*
11	CH	6^14∕05^	*Klebsiella* sp	*E. coli*
12	UBTH	3892	*Klebsiella* sp	*E. cloacae*
13	UBTH	3397	*Klebsiella* sp	*E. coli*
14	CH	7^14∕05^	*Klebsiella* sp	*E. coli*
15	UBTH	1468	*Klebsiella* sp	*E. coli*
16	UBTH	1443	*Klebsiella* sp	*E. coli*
17	UTBH	2570	*Klebsiella* sp	*E. coli*
18	UBTH	1476	*Klebsiella* sp	*E. cloacae*
19	IUTH	I2	*Klebsiella* sp	*E. cloacae*
20	IUTH	I1	*Klebsiella* sp	*E. cloacae*
21	UBTH	3978	*Klebsiella* sp	*E. coli*
22	UBTH	3385	*Klebsiella* sp	*E. coli*
23	UBTH	2695	*Klebsiella* sp	*E. coli*
24	UBTH	849	*Klebsiella* sp	*E. asburiae*
25	UBTH	2644	*Klebsiella* sp	*E. cloacae*
26	UBTH	570	*Klebsiella* sp	*E. coli*
27	UBTH	18	*Klebsiella* sp	*E. coli*
28	CH	K	*Klebsiella* sp	*E. cloacae*
29	UBTH	1356	*Klebsiella* sp	*E. coli*
30	UBTH	3628	*Klebsiella* sp	*E. cloacae*
31	UBTH	1120	*Klebsiella* sp	*S. marcescens*
32	UBTH	3647	*Klebsiella* sp	*S. marcescens*
33	UBTH	2697	*Klebsiella* sp	*E. coli*
34	UBTH	3599	*Klebsiella* sp	*S. marcescens*
35	UBTH	734(2)	*Klebsiella* sp	*E. asburiae*
36	UBTH	A^30∕04^	*E. coli*	*C. freundii*
37	CH	1123	*E. coli*	*S. marcescens*
38	UBTH	1148	*E. coli*	*S. marcescens*
39	UBTH	669	*E. coli*	*C. freundii*
40	UBTH	1628	*E. coli*	*K. pneumoniae*
41	UBTH	3337	*E. coli*	*L. adecarboxylata*
42	UBTH	2781	*E. coli*	*E. cloacae*
43	UBTH	872	*E. coli*	*K. pneumoniae*
44	UBTH	Q9	*E. coli*	*E. cloacae*
45	UBTH	14	*E. coli*	*K. pneumoniae*
46	UBTH	2580	*E. coli*	*C. freundii*
47	UBTH	656	*E. coli*	*K. pneumoniae*
48	UBTH	6145	*Citrobacter* sp	*E. coli*
49	UBTH	4641	*Citrobacter* sp	*E. asburiae*
50	UBTH	4507	*Citrobacter* sp	*E. cloacae*
51	UBTH	631	*Citrobacter* sp	*E. coli*
52	UBTH	3567	*Citrobacter* sp	*E. cloacae*
53	UBTH	2840	*Citrobacter* sp	*K. pneumoniae*
54	UBTH	885	*Citrobacter* sp	*K. pneumoniae*
55	UBTH	1678	*K. oxytoca*	*E. coli*
56	UBTH	2654	*K. oxytoca*	*E. cloacae*
57	UBTH	2845	*K. oxytoca*	*E. coli*
58	UBTH	2767LF	*K. oxytoca*	*E. asburiae*
59	UBTH	1677	*K. oxytoca*	*E. coli*
60	UBTH	3577	*P. vulgaris*	*P. rettgeri*
61	UBTH	1099	*P. vulgaris*	*M. morganii*
62	UBTH	3304	*P. vulgaris*	*M. morganii*
63	UBTH	5006	*Proteus* sp	*P. rettgeri*

## Results

One hundred and forty five (98.6%) isolates had a MALDI Biotyper best score > or =2.0, indicating a secure genus and probable species identification; and 2(1.36%) isolates (One *Enterobacter cloacae* and one *E. coli*) had a best score < 2.0 indicating probable genus identification. Isolates with Biotyper best scores of > or =2.0 comprised nine genera and 10 species respectively. A total of 57.2% and 33.1% of isolates identified were in agreement between the MALDI Biotyper and conventional identification at the genus level and species level respectively when analyzing bacteria with MALDI Biotyper best scores > or =2.0. Most of the identities (98.6%) obtained with MALDI-TOF Biotyper best scores were confirmed with second best scores hereby validating MALDI-TOF MS identification. The group *Klebsiella, Enterobacter, Serratia and Citrobacter* (KESC group) are closely related and there are often misidentifications within this group when using conventional methods to identify them. If these are considered as a group, the conventional identification method has a concordance of 75.2% compared to the MALDI-TOF MS identification method. Table 3 shows the conventional test identifications and its concordance on a genus and species level for the Enterobacteriaceae isolates. Among the 147 isolates, MALDI-TOF MS identified 35 isolates as *Klebsiella pneumoniae*. No isolate was identified as *K. pneumoniae* by the local identification methods. MALDI-TOF MS identified 71 isolates as *E. coli*, whereas the conventional methods detected 49 (69.0%) of these as *E. coli*. Four isolates were identified as *Citrobacter freundii* by MALDI-TOF MS and none of these isolates were detected by conventional methods. Twelve isolates identified as *E. coli* by conventional methods were identified as *K. pneumoniae* (*n* = 4), *C. freundii* (*n* = 3), *E. cloacae* (*n* = 2), *S. marcescens* (*n* = 2), *L. adecarboxylata* (*n* = 1) by the MALDI-TOF MS (Table 4). Thirty-five isolates identified as *Klebsiella* sp by conventional methods were identified either as *K. pneumoniae, E. coli, S. marcescens, C, freundii, E. asburiae* or *E. cloacae* by MALDI-TOF MS (Table 4). Five isolates identified as *K. oxytoca* and all *Citrobacter sp* isolates identified by conventional methods were identified either as *K. pneumoniae, E. coli, E. asburiae* or *E. cloacae* by the MALDI-TOF MS (Table 4). MALDI-TOF MS also identified six isolates as *Enterobacter asburiae*, four isolates as *Morganella morganii* and fifteen isolates as *Enterobacter cloacae* which were not identified by the local identification methods. Two isolates were identified as *Providencia rettgeri*, one isolate as *Leclercia adecarboxylata*, three isolates *as Proteus mirabilis* and six isolates as *Serratia marcescens* by MALDI-TOF MS, which were also not detected by the conventional methods. Isolates identified as *Proteus sp* and *P. vulgaris* by the conventional methods were identified either as *M. morganii*, *P. rettgeri* or *Proteus mirabilis* using MALDI-TOF MS (Table 4). Comparing the cost at a local level (e.g., Nigeria) of the conventional tests with MALDI-TOF MS was difficult to access, as the conventional methods for identification were carried out on all isolates in the three separate laboratories in the hospitals from where the isolates were obtained before analyzing them in this study with MALDI-TOF MS. Identification with MALDI-TOF MS was carried out in a separate laboratory different from where the conventional methods for identification were done.

## Discussion

MALDI-TOF MS has been successfully used for the identification of a wide array of bacteria (Mellmann et al., 2008; Saleeb et al., 2011; Panda et al., 2014; Ayeni et al., 2015). In developing countries such as Nigeria, prompt and accurate identification of microorganisms is a major challenge to medical microbiologists. Most medical microbiologists rely on conventional techniques (microscopic examination, for example the Gram staining test, culture on selective media and biochemical tests, for example catalase test, indole test) to identify microorganisms (Cheesebrough, 2000). Often, identification using conventional techniques is not very accurate, leading to misidentification of the microorganism. As a consequence, the treatment of patients is inappropriate (Ayeni et al., 2015). Only a few studies have assessed the performance of MALDI-TOF MS based identification versus conventional microbial identification techniques commonly used in developing countries (Iroha et al., 2011; Ayeni et al., 2015). In this study, a total of 57.2% and 33.1% of isolates identified were in agreement between MALDI-TOF MS and conventional methods for identification at genus and species level respectively when analyzing bacteria with MALDI Biotyper best scores > or =2.0. Discrepant results were not resolved using an additional bacterial identification method. Conventional methods require a large inoculum and often need subculturing for 24–48 h after culturing on a selective media which increases the turnaround time for identification thereby delaying prompt therapeutic intervention (Tan et al., 2012). Previous reports show that MALDI-TOF technique has a higher accuracy for most microbial identifications and performed equally or better than conventional techniques without a prior knowledge of the type of microorganism (Bader et al., 2011; Van Veen et al., 2010). In our study, a possible reason for misidentification of microorganisms may be an incomplete analysis using several biochemical tests before concluding on the identity of the isolates. As a result of the costs and time involved in carrying out such tests usually only a few basic tests are carried out which may in turn lead to misidentification of isolates. Previous reports show that the use of MALDI-TOF MS in clinical laboratories reduced the costs for hospitals in developed countries (Seng et al., 2009; Bizzini & Greub, 2010; Cherkaoui et al., 2010; Galloit O et al., 2011; Tan et al., 2012). The costs of bacterial identification by MALDI-TOF MS was estimated to be only 17%–32% (around €1.43/sample) of the costs of conventional identification methods €4.6–8.23/sample). In a previous study (El-Bouri et al., 2012), the cost for MALDI Biotyper identification was estimated as low as £0.51 per isolate which may compare more favourably with cost of conventional tests done in developing countries like Nigeria. However, the accuracy and reliability of results obtained by MALDI-TOF MS to allow prompt therapeutic intervention cannot be overemphasized. Initial set up cost for MALDI-TOF MS is expensive but running costs is minimal compared with conventional identification methods (Cherkaoui et al., 2010). The costs of MALDI-TOF MS instruments (purchase and maintenance) and the need of electricity to run the instruments may pose the main challenge of a general use in developing countries. However, overcoming the challenge of electric power supply in developing countries to incubate microbial cultures under appropriate temperature conditions could as well be applied to the running of MALDI-TOF MS instruments. Implementation of the use of MALDI-TOF MS in identifying clinical isolates in developing countries is possible, if local and concerned authorities realize the importance of accurately identifying bacterial isolates and its impact on public health. MALDI-TOF MS reagents are inexpensive, they do not expire and do not require specific storage conditions (Seng et al., 2009; Cherkaoui et al., 2010; Seng et al., 2013) compared to reagents used in conventional identification methods. The total cost of care should also be considered. Identifying the cause of an infection more quickly might be able to avoid additional costs such as admission costs as a result of long hospital stays, unnecessary antibiotic use etc. To get the best value of MALDI-TOF MS, it has to be used at its full capacity which can maximize resources and minimize costs. This can be achieved by batching several runs in a day and ensuring all sample spots on the target are filled before loading onto the MS. This should be possible without impacting the turnaround time significantly. Clinical records in the laboratory should also be effectively managed to achieve the best use of MALDI-TOF MS when used at full capacity.

The ability of MALDI-TOF MS to identify 98.6% of the isolates tested in this study to the species level based on log (score) of ≥2.0 underlines the considerable potential of this technique for bacterial species identification. The accuracy of identification was similar to that obtained in previous reports (Cherkaoui et al., 2010; Bizzini et al., 2011; Samb-Ba et al., 2014). Further research on applications of MALDI-TOF MS promises additional benefits for clinical microbiology.

## Conclusion

This study shows that owing to its rapid nature and specificity (accuracy of results) MALDI-TOF MS should replace conventional biochemical methods used in clinical medical microbiology settings, especially in developing countries like Nigeria. This is especially important as reliable results can be reported earlier for quick and accurate therapeutic intervention improving patient care.

##  Supplemental Information

10.7717/peerj.2511/supp-1 Supplemental Information 1 Data showing conventional test results of the isolates

10.7717/peerj.2511/supp-2 Supplemental Information 2 Summarized data of Best mean MALDI scores of Isolates

10.7717/peerj.2511/supp-3 Supplemental Information 3 Data comparing identification by conventional methods and MALDI-TOF MS

10.7717/peerj.2511/supp-4 Supplemental Information 4 Data of Enterobacteriaceae isolates provided by 3 Nigerian Hospitals

## Additional Information and Declarations

### Data Availability

The following information was supplied regarding data availability: The raw data has been supplied as Supplementary Files.
