# Supplementary material for: Accuracy of conventional identification methods used for Enterobacteriaceae isolates in three Nigerian hospitals
Source: PeerJ. 2016 Sep 28;4:e2511. doi: 10.7717/peerj.2511 (PMC5045884; doi:10.7717/peerj.2511)
Supplement: Supplemental Information 1 [file peerj-04-2511-s001.docx]

| **Number** | **Hospital** | **Identification** | **GST** | **M** | **B-GT** | **CUT** | **UT** | **INT** | **LFT** | **Conventional tests identification** |
| --- | --- | --- | --- | --- | --- | --- | --- | --- | --- | --- |
| 1 | UBTH | 6145 | -ve | sr | ND | +ve | ND | -ve | +ve | *Citrobacter sp* |
| 2 | UBTH | 5823 | -ve | sr | +ve | -ve | -ve | +ve | +ve | *e.coli* |
| 3 | UBTH | 1482 | -ve | r | ND | +ve | +ve | -ve | +ve | *Kleb sp* |
| 4 | UBTH | 1677 | -ve | r | ND | +ve | +ve | +ve | +ve | *K.oxytoca* |
| 5 | UBTH | 4641 | -ve | sr | ND | +ve | ND | -ve | +ve | *Citrobacter sp* |
| 6 | UBTH | 1643 | -ve | sr | +ve | -ve | -ve | +ve | +ve | *E.coli* |
| 7 | UBTH | 1670 | -ve | sr | +ve | -ve | -ve | +ve | +ve | *E.coli* |
| 8 | UBTH | 1678 | -ve | r | ND | +ve | +ve | +ve | +ve | *K.oxytoca* |
| 9 | UBTH | 1337 | -ve | r | ND | +ve | +ve | -ve | +ve | *Kleb sp* |
| 10 | UBTH | 5153 | -ve | sr | +ve | -ve | -ve | +ve | +ve | *E. coli* |
| 11 | UBTH | 1240 | -ve | r | ND | +ve | +ve | -ve | +ve | *Kleb sp* |
| 12 | UBTH | 4507 | -ve | sr | ND | +ve | ND | -ve | +ve | *Citrobacter sp* |
| 13 | UBTH | 5006 | -ve | r | ND | ND | +ve | -ve | -ve | *Proteus sp* |
| 14 | UBTH | 1453 | -ve | sr | +ve | -ve | -ve | +ve | +ve | *E.coli* |
| 15 | UBTH | 1259 | -ve | r | ND | +ve | +ve | -ve | +ve | *Kleb sp* |
| 16 | UBTH | 5832 | -ve | r | ND | +ve | +ve | -ve | +ve | *Kleb sp* |
| 17 | UBTH | 4501 | -ve | r | ND | +ve | +ve | -ve | +ve | *Kleb sp* |
| 18 | UBTH | 4354 | -ve | sr | +ve | -ve | -ve | +ve | +ve | *E.coli* |
| 19 | UBTH | 5854 | -ve | r | ND | +ve | +ve | -ve | +ve | *Kleb sp* |
| 20 | UBTH | 1628 | -ve | sr | +ve | -ve | -ve | +ve | +ve | *E.coli* |
| 21 | UBTH | 1681 | -ve | sr | +ve | -ve | -ve | +ve | +ve | *E.coli* |
| 22 | UBTH | 5774 | -ve | r | ND | +ve | +ve | -ve | +ve | *Kleb sp* |
| 23 | CH | G | -ve | sr | +ve | -ve | -ve | +ve | +ve | *E coli* |
| 24 | IUTH | 6II | -ve | r | ND | +ve | +ve | -ve | +ve | *Kleb sp* |
| 25 | UBTH | 669 | -ve | sr | +ve | -ve | -ve | +ve | +ve | *E.coli* |
| 26 | UBTH | 3520 | -ve | sr | +ve | -ve | -ve | +ve | +ve | *E.coli* |
| 27 | CH | C4 | -ve | r | ND | ND | +ve | -ve | -ve | *Proteus sp* |
| 28 | UBTH | 2 | -ve | sr | +ve | -ve | -ve | +ve | +ve | *E.coli* |
| 29 | UBTH | 573 | -ve | sr | +ve | -ve | -ve | +ve | +ve | *E.coli* |
| 30 | UBTH | 3467(2) | -ve | sr | +ve | -ve | -ve | +ve | +ve | *E.coli* |
| 31 | UBTH | 3461 | -ve | r | ND | +ve | +ve | -ve | +ve | *Kleb sp* |
| 32 | UBTH | 731 | -ve | r | ND | +ve | +ve | -ve | +ve | *Kleb sp* |
| 33 | UBTH | 138 | -ve | r | ND | +ve | +ve | -ve | +ve | *Kleb sp* |
| 34 | UBTH | 3600 | -ve | r | ND | +ve | +ve | -ve | +ve | *Kleb sp* |
| 35 | UBTH | 3324(12) | -ve | r | ND | ND | +ve | +ve | -ve | *P.vulgaris* |
| 36 | UBTH | 3682 | -ve | r | ND | +ve | +ve | -ve | +ve | *Kleb sp* |
| 37 | UBTH | 8(2) | -ve | r | ND | +ve | +ve | -ve | +ve | *Kleb sp* |
| 38 | UBTH | 3264 | -ve | r | ND | +ve | +ve | -ve | +ve | *Kleb sp* |
| 39 | UBTH | 1148 | -ve | sr | +ve | -ve | -ve | +ve | +ve | *E.coli* |
| 40 | UBTH | 1123 | -ve | sr | +ve | -ve | -ve | +ve | +ve | *E.coli* |
| 41 | UBTH | 3577 | -ve | r | ND | ND | +ve | +ve | -ve | *P.vulgaris* |
| 42 | IUTH | 20 | -ve | sr | +ve | -ve | -ve | +ve | +ve | *E.coli* |
| 43 | CH | A^30/04^ | -ve | sr | +ve | -ve | -ve | +ve | +ve | *E.coli* |
| 44 | CH | B^30/04^ | -ve | r | ND | ND | +ve | -ve | -ve | *Proteus sp* |
| 45 | CH | 8^14/05^ | -ve | r | ND | +ve | +ve | -ve | +ve | *Kleb sp* |
| 46 | UBTH | 1235 | -ve | sr | +ve | -ve | -ve | +ve | +ve | *E.coli* |
| 47 | UBTH | 3904 | -ve | r | ND | +ve | +ve | -ve | +ve | *Kleb sp* |
| 48 | CH | 6^14/05^ | -ve | r | ND | +ve | +ve | -ve | +ve | *Kleb sp* |
| 49 | UBTH | 3304 | -ve | r | ND | ND | +ve | +ve | -ve | *P.vulgaris* |
| 50 | UBTH | 263 | -ve | r | ND | +ve | +ve | -ve | +ve | *Kleb sp* |
| 51 | UBTH | 4595 | -ve | r | ND | +ve | +ve | -ve | +ve | *Kleb sp* |
| 52 | UBTH | 3892 | -ve | r | ND | +ve | +ve | -ve | +ve | *Kleb sp* |
| 53 | UBTH | 3337 | -ve | sr | +ve | -ve | -ve | +ve | +ve | *E.coli* |
| 54 | UBTH | 3397 | -ve | r | ND | +ve | +ve | -ve | +ve | *Kleb sp* |
| 55 | CH | 7^14/05^ | -ve | r | ND | +ve | +ve | -ve | +ve | *Kleb sp* |
| 56 | UBTH | 1468 | -ve | r | ND | +ve | +ve | -ve | +ve | *Kleb sp* |
| 57 | UBTH | 11 | -ve | sr | +ve | -ve | -ve | +ve | +ve | *E.coli* |
| 58 | UBTH | 1443 | -ve | r | ND | +ve | +ve | -ve | +ve | *Kleb sp* |
| 59 | IUTH | ADI | -ve | sr | +ve | -ve | -ve | +ve | +ve | *E.coli* |
| 60 | UBTH | 9(2) | -ve | r | ND | +ve | -ve | -ve | +ve | *Enterobacter sp* |
| 61 | CH | K^30/04^ | -ve | r | ND | ND | +ve | -ve | -ve | *Proteus sp* |
| 62 | UBTH | 2570 | -ve | r | ND | +ve | +ve | -ve | +ve | *Kleb sp* |
| 63 | UBTH | 3976 | -ve | sr | +ve | -ve | -ve | +ve | +ve | *E.coli* |
| 64 | UBTH | 1476 | -ve | r | ND | +ve | +ve | -ve | +ve | *Kleb sp* |
| 65 | UBTH | 1464 | -ve | sr | +ve | -ve | -ve | +ve | +ve | *E.coli* |
| 66 | UBTH | Y2 | -ve | sr | +ve | -ve | -ve | +ve | +ve | *E. coli* |
| 67 | IUTH | I2 | -ve | r | ND | +ve | +ve | -ve | +ve | *Kleb sp* |
| 68 | IUTH | I1 | -ve | r | ND | +ve | +ve | -ve | +ve | *Kleb sp* |
| 69 | UBTH | 3978 | -ve | r | ND | +ve | +ve | -ve | +ve | *Kleb sp* |
| 70 | CH | C30-04 | -ve | sr | +ve | -ve | -ve | +ve | +ve | *E.coli* |
| 71 | CH | F30-04 | -ve | r | ND | +ve | +ve | -ve | +ve | *Kleb sp* |
| 72 | CH | D30-04 | -ve | r | ND | +ve | +ve | -ve | +ve | *Kleb sp* |
| 73 | UBTH | 3385 | -ve | r | ND | +ve | +ve | -ve | +ve | *Kleb sp* |
| 74 | UBTH | 4113 | -ve | sr | +ve | -ve | -ve | +ve | +ve | *E.coli* |
| 75 | UBTH | 4349 | -ve | sr | +ve | -ve | -ve | +ve | +ve | *E.coli* |
| 76 | UBTH | 631 | -ve | sr | ND | +ve | ND | -ve | +ve | *Citrobacter sp* |
| 77 | UBTH | 837 | -ve | sr | +ve | -ve | -ve | +ve | +ve | *E.coli* |
| 78 | UBTH | 2767LF | -ve | r | ND | +ve | +ve | +ve | +ve | *K.oxytoca* |
| 79 | UBTH | Q5 | -ve | r | ND | +ve | +ve | -ve | +ve | *Kleb sp* |
| 80 | UBTH | 2845 | -ve | r | ND | +ve | +ve | +ve | +ve | *K.oxytoca* |
| 81 | UBTH | 2822 | -ve | r | ND | +ve | +ve | +ve | +ve | *K. oxytoca* |
| 82 | UBTH | 3611 | -ve | sr | +ve | -ve | -ve | +ve | +ve | *E.coli* |
| 83 | UBTH | 2821 | -ve | sr | +ve | -ve | -ve | +ve | +ve | *E.coli* |
| 84 | UBTH | 4374 | -ve | sr | +ve | -ve | -ve | +ve | +ve | *E.coli* |
| 85 | UBTH | 1333LF | -ve | sr | +ve | -ve | -ve | +ve | +ve | *E.coli* |
| 86 | UBTH | 4909 | -ve | sr | +ve | -ve | -ve | +ve | +ve | *E.coli* |
| 87 | UBTH | 4502 | -ve | r | ND | +ve | +ve | -ve | +ve | *Kleb sp* |
| 88 | UBTH | 2654 | -ve | r | ND | +ve | +ve | +ve | +ve | *K.oxytoca* |
| 89 | UBTH | 157 | -ve | r | ND | +ve | +ve | -ve | +ve | *Kleb sp* |
| 90 | UBTH | 4387 | -ve | r | ND | +ve | +ve | -ve | +ve | *Kleb sp* |
| 91 | UBTH | 2695 | -ve | r | ND | +ve | +ve | -ve | +ve | *Kleb sp* |
| 92 | UBTH | 1371 | -ve | sr | +ve | -ve | -ve | +ve | +ve | *E.coli* |
| 93 | UBTH | Q7 | -ve | sr | +ve | -ve | -ve | +ve | +ve | *E.coli* |
| 94 | UBTH | 878 | -ve | sr | +ve | -ve | -ve | +ve | +ve | *E.coli* |
| 95 | UBTH | 2781 | -ve | sr | +ve | -ve | -ve | +ve | +ve | *E.coli* |
| 96 | UBTH | 872 | -ve | sr | +ve | -ve | -ve | +ve | +ve | *E.coli* |
| 97 | UBTH | Q1 | -ve | r | ND | +ve | +ve | -ve | +ve | *Kleb sp* |
| 98 | UBTH | 2804 | -ve | sr | +ve | -ve | -ve | +ve | +ve | *e.coli* |
| 99 | UBTH | 849 | -ve | r | ND | +ve | +ve | -ve | +ve | *Kleb sp* |
| 100 | UBTH | Q8 | -ve | sr | +ve | -ve | -ve | +ve | +ve | *E. coli* |
| 101 | UBTH | 852 | -ve | r | ND | +ve | +ve | -ve | +ve | *Kleb sp* |
| 102 | UBTH | 1337LF | -ve | r | ND | +ve | +ve | +ve | +ve | *k.oxytoca* |
| 103 | UBTH | 2803 | -ve | sr | +ve | -ve | -ve | +ve | +ve | *E.coli* |
| 104 | UBTH | Q9 | -ve | sr | +ve | -ve | -ve | +ve | +ve | *E.coli* |
| 105 | UBTH | 2835 | -ve | sr | +ve | -ve | -ve | +ve | +ve | *E.coli* |
| 106 | UBTH | 3442 | -ve | sr | +ve | -ve | -ve | +ve | +ve | *E.coli* |
| 107 | UBTH | 2840 | -ve | sr | ND | +ve | ND | -ve | +ve | *Citrobacter sp* |
| 108 | UBTH | 885 | -ve | sr | ND | +ve | ND | -ve | +ve | *Citrobacter sp* |
| 109 | UBTH | 852K | -ve | r | ND | +ve | +ve | +ve | +ve | *K.oxytoca* |
| 110 | UBTH | 2833 | -ve | r | ND | +ve | +ve | +ve | +ve | *K.oxytoca* |
| 111 | UBTH | 6450 | -ve | sr | +ve | -ve | -ve | +ve | +ve | *E.coli* |
| 112 | UBTH | 2819 | -ve | sr | +ve | -ve | -ve | +ve | +ve | *E.coli* |
| 113 | UBTH | 1139 | -ve | sr | +ve | -ve | -ve | +ve | +ve | *E.coli* |
| 114 | UBTH | 2644 | -ve | r | ND | +ve | +ve | -ve | +ve | *Kleb sp* |
| 115 | UBTH | 2348 | -ve | sr | +ve | -ve | -ve | +ve | +ve | *E.coli* |
| 116 | CH | C2 | -ve | r | ND | +ve | +ve | -ve | +ve | *Kleb sp* |
| 117 | UBTH | 14 | -ve | sr | +ve | -ve | -ve | +ve | +ve | *E.coli* |
| 118 | UBTH | 570 | -ve | r | ND | +ve | +ve | -ve | +ve | *Kleb sp* |
| 119 | IUTH | 18 | -ve | r | ND | +ve | +ve | -ve | +ve | *Kleb sp* |
| 120 | UBTH | 2511 | -ve | r | ND | +ve | +ve | -ve | +ve | *Kleb sp* |
| 121 | CH | A3 | -ve | r | ND | +ve | +ve | -ve | +ve | *Kleb sp* |
| 122 | UBTH | 662 | -ve | sr | +ve | -ve | -ve | +ve | +ve | *E.coli* |
| 123 | IUTH | 19 | -ve | r | ND | +ve | +ve | -ve | +ve | *Kleb sp* |
| 124 | UBTH | 12 | -ve | sr | +ve | -ve | -ve | +ve | +ve | *E.coli* |
| 125 | UBTH | 764 | -ve | sr | +ve | -ve | -ve | +ve | +ve | *E.coli* |
| 126 | UBTH | 3567 | -ve | sr | ND | +ve | ND | -ve | +ve | *Citrobacter sp* |
| 127 | IUTH | 21 | -ve | sr | +ve | -ve | -ve | +ve | +ve | *E.coli* |
| 128 | UBTH | 627 | -ve | sr | +ve | -ve | -ve | +ve | +ve | *E.coli* |
| 129 | CH | K | -ve | r | ND | +ve | +ve | -ve | +ve | *Kleb sp* |
| 130 | CH | C8 | -ve | r | ND | +ve | +ve | -ve | +ve | *Kleb sp* |
| 131 | UBTH | 2471 | -ve | r | ND | +ve | +ve | -ve | +ve | *Kleb sp* |
| 132 | UBTH | OMIJIE | -ve | sr | +ve | -ve | -ve | +ve | +ve | *E.coli* |
| 133 | UBTH | 3471 | -ve | sr | +ve | -ve | -ve | +ve | +ve | *E.coli* |
| 134 | UBTH | 13 | -ve | sr | +ve | -ve | -ve | +ve | +ve | *E.coli* |
| 135 | UBTH | 2580 | -ve | sr | +ve | -ve | -ve | +ve | +ve | *E.coli* |
| 136 | UBTH | 734-2 | -ve | r | ND | +ve | +ve | -ve | +ve | *Kleb sp* |
| 137 | UBTH | UI | -ve | r | ND | +ve | +ve | -ve | +ve | *Kleb sp* |
| 138 | UBTH | 1099 | -ve | r | ND | ND | +ve | +ve | -ve | *P. vulgaris* |
| 139 | UBTH | 3599 | -ve | r | ND | +ve | +ve | -ve | +ve | *Kleb sp* |
| 140 | UBTH | 602 | -ve | r | ND | +ve | +ve | -ve | +ve | *Kleb sp* |
| 141 | UBTH | 2697 | -ve | r | ND | +ve | +ve | -ve | +ve | *Kleb sp* |
| 142 | UBTH | 3647 | -ve | r | ND | +ve | +ve | -ve | +ve | *Kleb sp* |
| 143 | UBTH | 846 | -ve | sr | +ve | -ve | -ve | +ve | +ve | *E.coli* |
| 144 | UBTH | 1120 | -ve | r | ND | +ve | +ve | -ve | +ve | *Kleb sp* |
| 145 | UBTH | 3628 | -ve | r | ND | +ve | +ve | -ve | +ve | *Kleb sp* |
| 146 | UBTH | 656 | -ve | sr | +ve | -ve | -ve | +ve | +ve | *E.coli* |
| 147 | UBTH | 1356 | -ve | r | ND | +ve | +ve | -ve | +ve | *Kleb sp* |
