# Supplementary material for: Accuracy of conventional identification methods used for Enterobacteriaceae isolates in three Nigerian hospitals
Source: PeerJ. 2016 Sep 28;4:e2511. doi: 10.7717/peerj.2511 (PMC5045884; doi:10.7717/peerj.2511)
Supplement: Supplemental Information 2 [file peerj-04-2511-s002.docx]

**Summarized Data of best mean MALDI-TOF MS log (score) identification values of 147 Enterobacteriaceae isolates**

| **Enterobacteriaceae isolates** | **Best mean score value** |
| --- | --- |
| *Escherichia coli* | 1.975-2.463 |
| *Klebsiella pneumoniae* | 2.101-2.558 |
| *Enterobacter asburiae* | 2.002-2.221 |
| *Enterobacter cloacae* | 1.952-2.325 |
| *Morganella morganii* | 2.324-2.538 |
| *Proteus mirabilis* | 2.127-2.452 |
| *Providencia rettgeri* | 2.023-2.41 |
| *C. freundii* | 2.164-2.322 |
| *Leclercia adecarboxylata*  *Serratia marcescens* | 2.006  2.153-2.331 |
|  |  |
