# Supplementary material for: Accuracy of conventional identification methods used for Enterobacteriaceae isolates in three Nigerian hospitals
Source: PeerJ. 2016 Sep 28;4:e2511. doi: 10.7717/peerj.2511 (PMC5045884; doi:10.7717/peerj.2511)
Supplement: Supplemental Information 3 [file peerj-04-2511-s003.docx]

| Number | Hospital | Identification | Conventional tests identification | **Maldi-tof identification (Best result)** | **Mean score value** | **Maldi-tof identification (second best result)** | **Mean score value** |
| --- | --- | --- | --- | --- | --- | --- | --- |
| 1 | UBTH | 6145 | *Citrobacter sp* | ***E.coli*** | **2.196** | ***E.coli*** | **2.136** |
| 2 | UBTH | 5823 | *e.coli* | ***E.coli*** | **2.318** | ***E.coli*** | **2.208** |
| 3 | UBTH | 1482 | *Kleb sp* | ***E.coli*** | **2.145** | ***E.coli*** | **2.038** |
| 4 | UBTH | 1677 | *K.oxytoca* | ***E.coli*** | **2.299** | ***E.coli*** | **2.229** |
| 5 | UBTH | 4641 | *Citrobacter sp* | ***Entr.asburiae*** | **2.221** | ***Entr.asburiae*** | **2.188** |
| 6 | UBTH | 1643 | *E.coli* | ***E.coli*** | **2.416** | ***E.coli*** | **2.324** |
| 7 | UBTH | 1670 | *E.coli* | ***E.coli*** | **2.283** | ***E.coli*** | **2.266** |
| 8 | UBTH | 1678 | *K.oxytoca* | ***E.coli*** | **2.195** | ***E.coli*** | **2.069** |
| 9 | UBTH | 1337 | *Kleb sp* | ***k.pneumoniae*** | **2.395** | ***k.pneumoniae*** | **2.306** |
| 10 | UBTH | 5153 | *E. coli* | ***E.coli*** | **2.213** | ***E.coli*** | **2.183** |
| 11 | UBTH | 1240 | *Kleb sp* | ***K.pneumoniae*** | **2.385** | ***K.pneumoniae*** | **2.269** |
| 12 | UBTH | 4507 | *Citrobacter sp* | ***Entr.cloacae*** | **1.952** | ***Entr.cloacae*** | **1.943** |
| 13 | UBTH | 5006 | *Proteus sp* | ***P.rettgeri*** | **2.41** | ***P.rettgeri*** | **2.285** |
| 14 | UBTH | 1453 | *E.coli* | ***E.coli*** | **2.421** | ***E.coli*** | **2.289** |
| 15 | UBTH | 1259 | *Kleb sp* | ***Entro.asburiae*** | **2.13** | ***Entro.asburiae*** | **2.048** |
| 16 | UBTH | 5832 | *Kleb sp* | ***Entero.asburiae*** | **2.11** | ***Entero.asburiae*** | **2.089** |
| 17 | UBTH | 4501 | *Kleb sp* | ***Entero.cloacae*** | **2.066** | ***Entero.cloacae*** | **2.031** |
| 18 | UBTH | 4354 | *E.coli* | ***E.coli*** | **2.386** | ***E.coli*** | **2.36** |
| 19 | UBTH | 5854 | *Kleb sp* | ***k.pneumoniae*** | **2.336** | ***k.pneumoniae*** | **2.284** |
| 20 | UBTH | 1628 | *E.coli* | ***k.pneumoniae*** | **2.387** | ***k.pneumoniae*** | **2.293** |
| 21 | UBTH | 1681 | *E.coli* | ***E.coli*** | **2.329** | ***E.coli*** | **2.297** |
| 22 | UBTH | 5774 | *Kleb sp* | ***E.cloacae*** | **2.272** | ***E.cloacae*** | **2.073** |
| 23 | CH | G | *E coli* | ***E.coli*** | **2.326** | ***E.coli*** | **2.253** |
| 24 | IUTH | 6II | *Kleb sp* | ***E.coli*** | **2.314** | ***E.coli*** | **2.283** |
| 25 | UBTH | 669 | *E.coli* | ***Citro.freundii*** | **2.178** | ***Citro.freundii*** | **2.17** |
| 26 | UBTH | 3520 | *E.coli* | ***E.coli*** | **2.218** | ***E.coli*** | **2.205** |
| 27 | CH | C4 | *Proteus sp* | ***P.mirabilis*** | **2.452** | ***P.mirabilis*** | **2.404** |
| 28 | UBTH | 2 | *E.coli* | ***E.coli*** | **2.437** | ***E.coli*** | **2.418** |
| 29 | UBTH | 573 | *E.coli* | ***E.coli*** | **2.232** | ***E.coli*** | **2.197** |
| 30 | UBTH | 3467(2) | *E.coli* | ***E.coli*** | **2.231** | ***E.coli*** | **2.127** |
| 31 | UBTH | 3461 | *Kleb sp* | ***C.freundii*** | **2.322** | ***C.freundii*** | **2.195** |
| 32 | UBTH | 731 | *Kleb sp* | ***k.pneumoniae*** | **2.548** | ***k.pneumoniae*** | **2.427** |
| 33 | UBTH | 138 | *Kleb sp* | ***S.marcescens*** | **2.153** | ***S.marcescens*** | **2.086** |
| 34 | UBTH | 3600 | *Kleb sp* | ***K.pneumoniae*** | **2.358** | ***K.pneumoniae*** | **2.345** |
| 35 | UBTH | 3324(12) | *P.vulgaris* | ***P.mirabilis*** | **2.136** | ***P.mirabilis*** | **2.105** |
| 36 | UBTH | 3682 | *Kleb sp* | ***K.pneumoniae*** | **2.459** | ***K.pneumoniae*** | **2.416** |
| 37 | UBTH | 8(2) | *Kleb sp* | ***E.coli*** | **2.394** | ***E.coli*** | **2.346** |
| 38 | UBTH | 3264 | *Kleb sp* | ***k.pneumoniae*** | **2.435** | ***k.pneumoniae*** | **2.373** |
| 39 | UBTH | 1148 | *E.coli* | ***S.marcescens*** | **2.181** | ***S.marcescens*** | **2.102** |
| 40 | UBTH | 1123 | *E.coli* | ***S.marcescens*** | **2.304** | ***S.marcescens*** | **2.263** |
| 41 | UBTH | 3577 | *P.vulgaris* | ***P.rettgeri*** | **2.023** | ***P.rettgeri*** | **2.002** |
| 42 | IUTH | 20 | *E.coli* | ***E.coli*** | **2.096** | ***E.coli*** | **2.051** |
| 43 | CH | A^30/04^ | *E.coli* | ***Citro.freundii*** | **2.277** | ***Citro.freundii*** | **2.249** |
| 44 | CH | B^30/04^ | *Proteus sp* | ***P.mirabilis*** | **2.127** | ***P.mirabilis*** | **2.102** |
| 45 | CH | 8^14/05^ | *Kleb sp* | ***K.pneumoniae*** | **2.344** | ***K.pneumoniae*** | **2.332** |
| 46 | UBTH | 1235 | *E.coli* | ***E.coli*** | **2.179** | ***E.coli*** | **2.039** |
| 47 | UBTH | 3904 | *Kleb sp* | ***Entr.cloacae*** | **2.095** | ***Entr.cloacae*** | **2.029** |
| 48 | CH | 6^14/05^ | *Kleb sp* | ***E.coli*** | **2.252** | ***E.coli*** | **2.227** |
| 49 | UBTH | 3304 | *P.vulgaris* | ***M.morganii*** | **2.538** | ***M.morganii*** | **2.319** |
| 50 | UBTH | 263 | *Kleb sp* | ***K.pneumoniae*** | **2.259** | ***K.pneumoniae*** | **2.199** |
| 51 | UBTH | 4595 | *Kleb sp* | ***k.pneumoniae*** | **2.354** | ***k.pneumoniae*** | **2.292** |
| 52 | UBTH | 3892 | *Kleb sp* | ***Entro.cloacae*** | **2.19** | ***Entro.cloacae*** | **2.17** |
| 53 | UBTH | 3337 | *E.coli* | ***Leclercia adecarboxylata*** | **2.006** | ***Leclercia adecarboxylata*** | **1.92** |
| 54 | UBTH | 3397 | *Kleb sp* | ***E.coli*** | **2.302** | ***E.coli*** | **2.275** |
| 55 | CH | 7^14/05^ | *Kleb sp* | ***E.coli*** | **2.274** | ***E.coli*** | **2.191** |
| 56 | UBTH | 1468 | *Kleb sp* | ***E.coli*** | **2.336** | ***E.coli*** | **2.302** |
| 57 | UBTH | 11 | *E.coli* | ***E.coli*** | **2.256** | ***E.coli*** | **2.219** |
| 58 | UBTH | 1443 | *Kleb sp* | ***E.coli*** | **2.249** | ***E.coli*** | **2.182** |
| 59 | IUTH | ADI | *E.coli* | ***E.coli*** | **2.215** | ***E.coli*** | **2.124** |
| 60 | UBTH | 9(2) | *Enterobacter sp* | ***Entr.cloacae*** | **2.126** | ***Entr.cloacae*** | **2.047** |
| 61 | CH | K^30/04^ | *Proteus sp* | ***P. mirablis*** | **2.227** | ***P. mirablis*** | **2.223** |
| 62 | UBTH | 2570 | *Kleb sp* | ***E.coli*** | **2.207** | ***E.coli*** | **2.198** |
| 63 | UBTH | 3976 | *E.coli* | ***E.coli*** | **2.358** | ***E.coli*** | **2.329** |
| 64 | UBTH | 1476 | *Kleb sp* | ***Entr.cloacae*** | **2.21** | ***Entr.cloacae*** | **2.154** |
| 65 | UBTH | 1464 | *E.coli* | ***E.coli*** | **2.157** | ***E.coli*** | **2.148** |
| 66 | UBTH | Y2 | *E. coli* | ***E. coli*** | **1.975** | ***E. coli*** | **1.71** |
| 67 | IUTH | I2 | *Kleb sp* | ***E.cloacae*** | **2.158** | ***E.cloacae*** | **2.101** |
| 68 | IUTH | I1 | *Kleb sp* | ***Entr.cloacae*** | **2.325** | ***Entr.cloacae*** | **2.117** |
| 69 | UBTH | 3978 | *Kleb sp* | ***E.coli*** | **2.236** | ***E.coli*** | **2.224** |
| 70 | CH | C30-04 | *E.coli* | ***E.coli*** | **2.386** | ***E.coli*** | **2.36** |
| 71 | CH | F30-04 | *Kleb sp* | ***K.pneumoniae*** | **2.136** | ***K.pneumoniae*** | **2.135** |
| 72 | CH | D30-04 | *Kleb sp* | ***K.pneumoniae*** | **2.254** | ***K.pneumoniae*** | **2.245** |
| 73 | UBTH | 3385 | *Kleb sp* | ***E.coli*** | **2.337** | ***E.coli*** | **2.316** |
| 74 | UBTH | 4113 | *E.coli* | ***E.coli*** | **2.308** | ***E.coli*** | **2.285** |
| 75 | UBTH | 4349 | *E.coli* | ***E.coli*** | **2.252** | ***E.coli*** | **2.033** |
| 76 | UBTH | 631 | *Citrobacter sp* | ***E.coli*** | **2.265** | ***E.coli*** | **2.226** |
| 77 | UBTH | 837 | *E.coli* | ***E.coli*** | **2.187** | ***E.coli*** | **2.05** |
| 78 | UBTH | 2767LF | *K.oxytoca* | ***E.asburiae*** | **2.109** | ***E.cloacae*** | **2.089** |
| 79 | UBTH | Q5 | *Kleb sp* | ***K.pneumoniae*** | **2.274** | ***K.pneumoniae*** | **2.272** |
| 80 | UBTH | 2845 | *K.oxytoca* | ***E.coli*** | **2.066** | ***E.coli*** | **2.049** |
| 81 | UBTH | 2822 | *K. oxytoca* | ***k.pneumoniae*** | **2.353** | ***k.pneumoniae*** | **2.343** |
| 82 | UBTH | 3611 | *E.coli* | ***E.coli*** | **2.35** | ***E.coli*** | **2.293** |
| 83 | UBTH | 2821 | *E.coli* | ***E.coli*** | **2.336** | ***E.coli*** | **2.243** |
| 84 | UBTH | 4374 | *E.coli* | ***E.coli*** | **2.257** | ***E.coli*** | **2.177** |
| 85 | UBTH | 1333LF | *E.coli* | ***E.coli*** | **2.352** | ***E.coli*** | **2.319** |
| 86 | UBTH | 4909 | *E.coli* | ***E.coli*** | **2.179** | ***E.coli*** | **2.139** |
| 87 | UBTH | 4502 | *Kleb sp* | ***K.pneumoniae*** | **2.365** | ***K.pneumoniae*** | **2.281** |
| 88 | UBTH | 2654 | *K.oxytoca* | ***E.cloacae*** | **2.126** | ***E.cloacae*** | **2.014** |
| 89 | UBTH | 157 | *Kleb sp* | ***K.pneumoniae*** | **2.363** | ***K.pneumoniae*** | **2.296** |
| 90 | UBTH | 4387 | *Kleb sp* | ***K.pneumoniae*** | **2.101** | ***K.pneumoniae*** | **2.038** |
| 91 | UBTH | 2695 | *Kleb sp* | ***E.coli*** | **2.217** | ***E.coli*** | **2.189** |
| 92 | UBTH | 1371 | *E.coli* | ***E.coli*** | **2.235** | ***E.coli*** | **2.164** |
| 93 | UBTH | Q7 | *E.coli* | ***E.coli*** | **2.222** | ***E.coli*** | **2.156** |
| 94 | UBTH | 878 | *E.coli* | ***E.coli*** | **2.308** | ***E.coli*** | **2.261** |
| 95 | UBTH | 2781 | *E.coli* | ***E.cloacae*** | **2.084** | ***E.cloacae*** | **2.065** |
| 96 | UBTH | 872 | *E.coli* | ***K.pneumoniae*** | **2.14** | ***K.pneumoniae*** | **2.006** |
| 97 | UBTH | Q1 | *Kleb sp* | ***K.pneumoniae*** | **2.405** | ***K.pneumoniae*** | **2.358** |
| 98 | UBTH | 2804 | *e.coli* | ***E.coli*** | **2.255** | ***E.coli*** | **2.152** |
| 99 | UBTH | 849 | *Kleb sp* | ***E.asburiae*** | **2.002** | ***E.cloacae*** | **1.91** |
| 100 | UBTH | Q8 | *E. coli* | ***E. coli*** | **2.254** | ***E. coli*** | **2.023** |
| 101 | UBTH | 852 | *Kleb sp* | ***K.pneumoniae*** | **2.351** | ***K.pneumoniae*** | **2.322** |
| 102 | UBTH | 1337LF | *k.oxytoca* | ***K.pneumoniae*** | **2.361** | ***K.pneumoniae*** | **2.351** |
| 103 | UBTH | 2803 | *E.coli* | ***E.coli*** | **2.266** | ***E.coli*** | **2.156** |
| 104 | UBTH | Q9 | *E.coli* | ***E.cloacae*** | **2.226** | ***E.cloacae*** | **2.128** |
| 105 | UBTH | 2835 | *E.coli* | ***E.coli*** | **2.159** | ***E.coli*** | **1.978** |
| 106 | UBTH | 3442 | *E.coli* | ***E.coli*** | **2.3** | ***E.coli*** | **2.15** |
| 107 | UBTH | 2840 | *Citrobacter sp* | ***K.pneumoniae*** | **2.371** | ***K.pneumoniae*** | **2.36** |
| 108 | UBTH | 885 | *Citrobacter sp* | ***K.pneumoniae*** | **2.292** | ***K.pneumoniae*** | **2.236** |
| 109 | UBTH | 852K | *K.oxytoca* | ***K.pneumoniae*** | **2.377** | ***K.pneumoniae*** | **2.32** |
| 110 | UBTH | 2833 | *K.oxytoca* | ***K.pneumoniae*** | **2.293** | ***K.pneumoniae*** | **2.146** |
| 111 | UBTH | 6450 | *E.coli* | ***E.coli*** | **2.137** | ***E.coli*** | **2.027** |
| 112 | UBTH | 2819 | *E.coli* | ***E.coli*** | **2.212** | ***E.coli*** | **2.072** |
| 113 | UBTH | 1139 | *E.coli* | ***E.coli*** | **2.224** | ***E.coli*** | **2.214** |
| 114 | UBTH | 2644 | *Kleb sp* | ***E. cloacae*** | **2.315** | ***E. cloacae*** | **2.307** |
| 115 | UBTH | 2348 | *E.coli* | ***E.coli*** | **2.122** | ***E.coli*** | **2.104** |
| 116 | CH | C2 | *Kleb sp* | ***K.pneumoniae*** | **2.424** | ***K.pneumoniae*** | **2.363** |
| 117 | UBTH | 14 | *E.coli* | ***K.pneumoniae*** | **2.287** | ***K.pneumoniae*** | **2.21** |
| 118 | UBTH | 570 | *Kleb sp* | ***E.coli*** | **2.288** | ***E.coli*** | **2.262** |
| 119 | IUTH | 18 | *Kleb sp* | ***E.coli*** | **2.186** | ***E.coli*** | **2.185** |
| 120 | UBTH | 2511 | *Kleb sp* | ***K.pneumoniae*** | **2.507** | ***K.pneumoniae*** | **2.409** |
| 121 | CH | A3 | *Kleb sp* | ***k.pneumoniae*** | **2.385** | ***k.pneumoniae*** | **2.371** |
| 122 | UBTH | 662 | *E.coli* | ***E.coli*** | **2.172** | ***E.coli*** | **2.093** |
| 123 | IUTH | 19 | *Kleb sp* | ***k.pneumoniae*** | **2.558** | ***k.pneumoniae*** | **2.546** |
| 124 | UBTH | 12 | *E.coli* | ***E.coli*** | **2.291** | ***E.coli*** | **2.277** |
| 125 | UBTH | 764 | *E.coli* | ***E.coli*** | **2.463** | ***E.coli*** | **2.32** |
| 126 | UBTH | 3567 | *Citrobacter sp* | ***M. morganii*** | **2.239** | ***M. morganii*** | **2.093** |
| 127 | IUTH | 21 | *E.coli* | ***E.coli*** | **2.312** | ***E.coli*** | **2.23** |
| 128 | UBTH | 627 | *E.coli* | ***E.coli*** | **2.405** | ***E.coli*** | **2.206** |
| 129 | CH | K | *Kleb sp* | ***E.cloacae*** | **2.288** | ***E.cloacae*** | **2.182** |
| 130 | CH | C8 | *Kleb sp* | ***K.pneumoniae*** | **2.515** | ***K.pneumoniae*** | **2.433** |
| 131 | UBTH | 2471 | *Kleb sp* | ***K.pneumoniae*** | **2.331** | ***K.pneumoniae*** | **2.276** |
| 132 | UBTH | OMIJIE | *E.coli* | ***E.coli*** | **2.038** | ***E.coli*** | **1.82** |
| 133 | UBTH | 3471 | *E.coli* | ***E.coli*** | **2.204** | ***E.coli*** | **2.01** |
| 134 | UBTH | 13 | *E.coli* | ***E.coli*** | **2.316** | ***E.coli*** | **2.305** |
| 135 | UBTH | 2580 | *E.coli* | ***C.freundii*** | **2.164** | ***C.freundii*** | **2.11** |
| 136 | UBTH | 734-2 | *Kleb sp* | ***E.asburiae*** | **2.149** | ***E.asburiae*** | **2.034** |
| 137 | UBTH | UI | *Kleb sp* | ***K.pneumoniae*** | **2.479** | ***K.pneumoniae*** | **2.344** |
| 138 | UBTH | 1099 | *P. vulgaris* | ***M. morganii*** | **2.324** | ***M. morganii*** | **2.18** |
| 139 | UBTH | 3599 | *Kleb sp* | ***S.marcescens*** | **2.331** | ***S.marcescens*** | **2.329** |
| 140 | UBTH | 602 | *Kleb sp* | ***k.pneumoniae*** | **2.37** | ***k.pneumoniae*** | **2.352** |
| 141 | UBTH | 2697 | *Kleb sp* | ***E.coli*** | **2.152** | ***E.coli*** | **2.097** |
| 142 | UBTH | 3647 | *Kleb sp* | ***S.marcescens*** | **2.239** | ***S.marcescens*** | **2.216** |
| 143 | UBTH | 846 | *E.coli* | ***E.coli*** | **2.254** | ***E.coli*** | **2.1** |
| 144 | UBTH | 1120 | *Kleb sp* | ***S. marcescens*** | **2.235** | ***S. marcescens*** | **2.031** |
| 145 | UBTH | 3628 | *Kleb sp* | ***E.cloacae*** | **2.193** | ***E.cloacae*** | **2.147** |
| 146 | UBTH | 656 | *E.coli* | ***K.pneumoniae*** | **2.156** | ***K.pneumoniae*** | **2.111** |
| 147 | UBTH | 1356 | *Kleb sp* | ***E.coli*** | **2.432** | ***E.coli*** | **2.404** |
