# Supplementary material for: Accuracy of conventional identification methods used for Enterobacteriaceae isolates in three Nigerian hospitals
Source: PeerJ. 2016 Sep 28;4:e2511. doi: 10.7717/peerj.2511 (PMC5045884; doi:10.7717/peerj.2511)
Supplement: Supplemental Information 4 [file peerj-04-2511-s004.docx]

| Number | Hospital | Identification | Conventional tests identification | Source | Sex | Age |
| --- | --- | --- | --- | --- | --- | --- |
| 1 | UBTH | 6145 | *Citrobacter sp* | URINE | M | 90 |
| 2 | UBTH | 5823 | *e.coli* | URINE | F | 58 |
| 3 | UBTH | 1482 | *Kleb sp* | URINE | M | AD |
| 4 | UBTH | 1677 | *K.oxytoca* | URINE | F | 23 |
| 5 | UBTH | 4641 | *Citrobacter sp* | URINE | M | AD |
| 6 | UBTH | 1643 | *E.coli* | URINE | F | 78 |
| 7 | UBTH | 1670 | *E.coli* | URINE | F | 63 |
| 8 | UBTH | 1678 | *K.oxytoca* | URINE | M | 30 |
| 9 | UBTH | 1337 | *Kleb sp* | SWAB | M | AD |
| 10 | UBTH | 5153 | *E. coli* | URINE | F | AD |
| 11 | UBTH | 1240 | *Kleb sp* | URINE | F | AD |
| 12 | UBTH | 4507 | *Citrobacter sp* | PLEURAL ASPIRATE | M | 38 |
| 13 | UBTH | 5006 | *Proteus sp* | WOUND SWAB | F | 36 |
| 14 | UBTH | 1453 | *E.coli* | URINE | M | 55 |
| 15 | UBTH | 1259 | *Kleb sp* | URINE | F | 57 |
| 16 | UBTH | 5832 | *Kleb sp* | URINE | F | 81 |
| 17 | UBTH | 4501 | *Kleb sp* | URINE | F | 73 |
| 18 | UBTH | 4354 | *E.coli* | URINE | F | 29 |
| 19 | UBTH | 5854 | *Kleb sp* | URINE | M | AD |
| 20 | UBTH | 1628 | *E.coli* | URINE | F | 15 |
| 21 | UBTH | 1681 | *E.coli* | URINE | F | AD |
| 22 | UBTH | 5774 | *Kleb sp* | SWAB | M | 32 |
| 23 | CH | G | *E coli* | HVS | M | 25 |
| 24 | IUTH | 6II | *Kleb sp* | URINE | F | 28 |
| 25 | UBTH | 669 | *E.coli* | EAR SWAB | M | 22 |
| 26 | UBTH | 3520 | *E.coli* | URINE | F |  |
| 27 | CH | C4 | *Proteus sp* | URINE | M | 46 |
| 28 | UBTH | 2 | *E.coli* | EAR SWAB | F | AD |
| 29 | UBTH | 573 | *E.coli* | ECS | F | 43 |
| 30 | UBTH | 3467(2) | *E.coli* | URINE | M | 60 |
| 31 | UBTH | 3461 | *Kleb sp* | EAR SWAB | F | 61 |
| 32 | UBTH | 731 | *Kleb sp* | URINE | M | AD |
| 33 | UBTH | 138 | *Kleb sp* | BLOOD CULTURE | M | 4 DAYS |
| 34 | UBTH | 3600 | *Kleb sp* | URINE | M | 33 |
| 35 | UBTH | 3324(12) | *P.vulgaris* | URINE | F | AD |
| 36 | UBTH | 3682 | *Kleb sp* | URINE | M | 72 |
| 37 | UBTH | 8(2) | *Kleb sp* | URINE | F | 35 |
| 38 | UBTH | 3264 | *Kleb sp* | URINE | F | AD |
| 39 | UBTH | 1148 | *E.coli* | URINE | F | 9 |
| 40 | UBTH | 1123 | *E.coli* | THROAT SWAB | M | AD |
| 41 | UBTH | 3577 | *P.vulgaris* | SYNOVIAL FLUID | F | 22 |
| 42 | IUTH | 20 | *E.coli* | HVS | F | 20 |
| 43 | CH | A^30/04^ | *E.coli* | URINE | F | AD |
| 44 | CH | B^30/04^ | *Proteus sp* | URINE | F | 20 |
| 45 | CH | 8^14/05^ | *Kleb sp* | URINE | F | 19 |
| 46 | UBTH | 1235 | *E.coli* | URINE | M | 52 |
| 47 | UBTH | 3904 | *Kleb sp* | WOUND SWAB | F | AD |
| 48 | CH | 6^14/05^ | *Kleb sp* | PUS | M | 60 |
| 49 | UBTH | 3304 | *P.vulgaris* | WOUND SWAB | F | AD |
| 50 | UBTH | 263 | *Kleb sp* | BLOOD | F | 5 DAYS |
| 51 | UBTH | 4595 | *Kleb sp* | URINE | F | AD |
| 52 | UBTH | 3892 | *Kleb sp* | UMBLICAL SWAB | M | 4 DAYS |
| 53 | UBTH | 3337 | *E.coli* | WOUND SWAB | M |  |
| 54 | UBTH | 3397 | *Kleb sp* | WOUND SWAB | F | AD |
| 55 | CH | 7^14/05^ | *Kleb sp* | URINE | F | 15 |
| 56 | UBTH | 1468 | *Kleb sp* | WOUND SWAB | M | AD |
| 57 | UBTH | 11 | *E.coli* | URINE | M | AD |
| 58 | UBTH | 1443 | *Kleb sp* | URINE | F | 25 |
| 59 | IUTH | ADI | *E.coli* | URINE | F | AD |
| 60 | UBTH | 9(2) | *Enterobacter sp* | URINE | F | 55 |
| 61 | CH | K^30/04^ | *Proteus sp* | URINE | F | 42 |
| 62 | UBTH | 2570 | *Kleb sp* | URINE | M | 43 |
| 63 | UBTH | 3976 | *E.coli* | URINE | F | 51 |
| 64 | UBTH | 1476 | *Kleb sp* | URINE | M | 63 |
| 65 | UBTH | 1464 | *E.coli* | URINE | M | 66 |
| 66 | UBTH | Y2 | *E. coli* | UIS |  |  |
| 67 | IUTH | I2 | *Kleb sp* | W SWAB | M | 32 |
| 68 | IUTH | I1 | *Kleb sp* | URINE | F | 28 |
| 69 | UBTH | 3978 | *Kleb sp* | URINE | M | 59 |
| 70 | CH | C30-04 | *E.coli* | HVS | F | 45 |
| 71 | CH | F30-04 | *Kleb sp* | HVS | F | 25 |
| 72 | CH | D30-04 | *Kleb sp* | WOUND | M | 62 |
| 73 | UBTH | 3385 | *Kleb sp* | CATHETER TIP | F | 58 |
| 74 | UBTH | 4113 | *E.coli* | URINE | F | 41 |
| 75 | UBTH | 4349 | *E.coli* | URINE | F | AD |
| 76 | UBTH | 631 | *Citrobacter sp* | URINE | F | AD |
| 77 | UBTH | 837 | *E.coli* | URINE | F | AD |
| 78 | UBTH | 2767LF | *K.oxytoca* | ECS | F | AD |
| 79 | UBTH | Q5 | *Kleb sp* | UIS |  |  |
| 80 | UBTH | 2845 | *K.oxytoca* | URINE | F | AD |
| 81 | UBTH | 2822 | *K. oxytoca* | WOUND SWAB | M | 7 mths |
| 82 | UBTH | 3611 | *E.coli* | URINE | F | 25 |
| 83 | UBTH | 2821 | *E.coli* | CATHETER TIP | M | 45 |
| 84 | UBTH | 4374 | *E.coli* | ECS |  |  |
| 85 | UBTH | 1333LF | *E.coli* | URINE | M | 50 |
| 86 | UBTH | 4909 | *E.coli* | URINE | F | 30 |
| 87 | UBTH | 4502 | *Kleb sp* | WOUND SWAB | M | AD |
| 88 | UBTH | 2654 | *K.oxytoca* | URINE | M | AD |
| 89 | UBTH | 157 | *Kleb sp* | BLOOD | M | 5 DAYS |
| 90 | UBTH | 4387 | *Kleb sp* | URINE | M | 75 |
| 91 | UBTH | 2695 | *Kleb sp* | WOUND SWAB | M | 40 |
| 92 | UBTH | 1371 | *E.coli* | URINE | F | 27 |
| 93 | UBTH | Q7 | *E.coli* | UIS |  |  |
| 94 | UBTH | 878 | *E.coli* | HVS | F | AD |
| 95 | UBTH | 2781 | *E.coli* | URINE | F | 28 |
| 96 | UBTH | 872 | *E.coli* | ECS | F | 46 |
| 97 | UBTH | Q1 | *Kleb sp* | UIS |  |  |
| 98 | UBTH | 2804 | *e.coli* | URINE | F | AD |
| 99 | UBTH | 849 | *Kleb sp* | W HVS ASPI | F | 55 |
| 100 | UBTH | Q8 | *E. coli* | UIS |  |  |
| 101 | UBTH | 852 | *Kleb sp* | URINE | M | 28 |
| 102 | UBTH | 1337LF | *k.oxytoca* | URINE | M | 56 |
| 103 | UBTH | 2803 | *E.coli* | URINE | M | 64 |
| 104 | UBTH | Q9 | *E.coli* | UIS |  |  |
| 105 | UBTH | 2835 | *E.coli* | WOUND SWAB | M | AD |
| 106 | UBTH | 3442 | *E.coli* | URINE | M | AD |
| 107 | UBTH | 2840 | *Citrobacter sp* | URINE | F | AD |
| 108 | UBTH | 885 | *Citrobacter sp* | URINE |  |  |
| 109 | UBTH | 852K | *K.oxytoca* | URINE | M | 28 |
| 110 | UBTH | 2833 | *K.oxytoca* | WOUND SWAB | F | 32 |
| 111 | UBTH | 6450 | *E.coli* | CATHETER TIP | F | 51 |
| 112 | UBTH | 2819 | *E.coli* | URINE | F | 65 |
| 113 | UBTH | 1139 | *E.coli* | URINE | M | 87 |
| 114 | UBTH | 2644 | *Kleb sp* | URINE | M | 60 |
| 115 | UBTH | 2348 | *E.coli* | URINE | F | AD |
| 116 | CH | C2 | *Kleb sp* | URINE | F | 28 |
| 117 | UBTH | 14 | *E.coli* | URINE | F | 52 |
| 118 | UBTH | 570 | *Kleb sp* | URINE | M | 70 |
| 119 | IUTH | 18 | *Kleb sp* | STOOL | F | 3 |
| 120 | UBTH | 2511 | *Kleb sp* | URINE | M | 67 |
| 121 | CH | A3 | *Kleb sp* | WOUND SWAB | F | 70 |
| 122 | UBTH | 662 | *E.coli* | URINE | F | 28 |
| 123 | IUTH | 19 | *Kleb sp* | WOUND SWAB | F | 31 |
| 124 | UBTH | 12 | *E.coli* | URINE | M | AD |
| 125 | UBTH | 764 | *E.coli* | EAR SWAB | M | AD |
| 126 | UBTH | 3567 | *Citrobacter sp* | URINE | F | AD |
| 127 | IUTH | 21 | *E.coli* | URINE | F | 20 |
| 128 | UBTH | 627 | *E.coli* | ECS | F | AD |
| 129 | CH | K | *Kleb sp* | URINE | F | 15 |
| 130 | CH | C8 | *Kleb sp* | URINE | F | 27 |
| 131 | UBTH | 2471 | *Kleb sp* | URINE | M | 62 |
| 132 | UBTH | OMIJIE | *E.coli* | URINE | F | AD |
| 133 | UBTH | 3471 | *E.coli* | URINE | F | 53 |
| 134 | UBTH | 13 | *E.coli* | URINE | F | AD |
| 135 | UBTH | 2580 | *E.coli* | URINE | F | 76 |
| 136 | UBTH | 734-2 | *Kleb sp* | URINE | M | 56 |
| 137 | UBTH | UI | *Kleb sp* | URINE | F | 33 |
| 138 | UBTH | 1099 | *P. vulgaris* | URINE | F | Ad |
| 139 | UBTH | 3599 | *Kleb sp* | URINE | M | 35 |
| 140 | UBTH | 602 | *Kleb sp* | URINE | F | 2 |
| 141 | UBTH | 2697 | *Kleb sp* | URINE | F | AD |
| 142 | UBTH | 3647 | *Kleb sp* | URINE | F | AD |
| 143 | UBTH | 846 | *E.coli* | URINE | F | AD |
| 144 | UBTH | 1120 | *Kleb sp* | WOUND SWAB | M | AD |
| 145 | UBTH | 3628 | *Kleb sp* | URINE | M | AD |
| 146 | UBTH | 656 | *E.coli* | ECS | F | 21 |
| 147 | UBTH | 1356 | *Kleb sp* | Urine | F | Ad |

ECS- Endocervical swab, UIS- Unidentified source, HVS- High vaginal swab, W HVS ASPI- Wound High vaginal swab aspirate
